# Editorial: Engineering Signal Sensors Based on Reprogrammed CRISPR Technologies

**DOI:** 10.3389/fmolb.2021.742961

**Published:** 2021-09-09

**Authors:** Yuchen Liu, Tao Xu, Yonghao Zhan

**Affiliations:** ^1^ Institute of Translational Medicine, Shenzhen Second People’s Hospital, First Affiliated Hospital of Shenzhen University, Shenzhen, China; ^2^ School of Pharmacy, Anhui Medical University, Hefei, China; ^3^ Department of Urology, The First Affiliated Hospital of Zhengzhou University, Zhengzhou, China

**Keywords:** CRISPR, SgRNA, signal sensor, disease diagnosis, disease treatment

In the past few years, gene editing and regulation technologies based on the CRISPR-Cas system have developed rapidly. Using CRISPR technology, we can not only knock out genes and edit DNA sequences, but also regulate DNA transcriptions, and even target RNAs. The expression of CRISPR-Cas system in cells includes both constitutive and inducible manners. The signal-inducible CRISPR-Cas system has the characteristics of temporal and spatial specific expression, so it can be used in medical research. This Research Topic aims to develop the reprogrammed CRISPR systems for sensing and targeting specific disease signals. It includes original research articles from communities involved with gene engineering, synthetic biology, RNA biology and tumor biology.

Firstly, as a gene editing and regulation tool, CRISPR-Cas can be used for cleaving DNA targets or regulating DNA transcription. By targeting specific disease markers, the CRISPR system can be used to study the functions and mechanisms of key molecules, and also directly inhibit the occurrence and development of diseases. Chen et al. used CRISPR-Cas9 system to knockout the HBV integrated fragments in PLC/PRF/5 cells that can potentially express HBsAg, which suggested the prospect of controlling hepatitis B virus replication and treating hepatitis Lan et al. revealed the possibility of using the CRISPR-dCas9 transcriptional suppression system to treat intervertebral disk degeneration (IDD) through Parkin targeted inhibition. Ye et al. used CRISPR-dCas9 to knockdown the expression of SNHG9, a lncRNA which promotes cell proliferation, migration, and invasion of human hepatocellular carcinoma cells by increasing GSTP1 methylation. By targeting the oncogenic lncRNA PANDAR, Jia et al. observed proliferation inhibition and apoptosis induction in oral squamous cell carcinoma using CRISPR-dCas9. Cao et al. also observed anticancer effects in bladder cancer by using a similar CRISPR-dCas9 system and indicated that lncRNA SNGH3 serves as a oncogene and could be employed as a prospective diagnostic marker for clinical use. Zhao et al. developed the CRISPR-dCas9-KRAB, a much more stronger transcriptional inhibitor, to regualte lncRNA gadd7 which protects spermatocyte viability. Zheng and Chen found that downregulation of CacyBP by CRISPR/dCas9-KRAB prevents bladder cancer progression and suggested that CacyBP is an important oncogene contributing to malignant behavior. Chen and Zheng reported that lncRNA LINC00944 promotes tumorigenesis but suppresses Akt phosphorylation in renal cell carcinoma by knockdown of LINC00944 using CRISPR-dCas9-KRAB. Yang et al. constructed the CRISPR-dCas9-VPR, a transcriptional activator, to up-regulate lncRNA ERIC exrepssion which inhibits cell proliferation and invasion and promotes apoptosis in human bladder cancer. Yang et al. used both CRISPR-dCas9-VPR and CRISPR-dCas9-KRAB systems to study the biological functions of ITGB5, TIMP1, and TMEM176B, and found that the three genes synergistically affect the proliferation, invasion and migration capabilities of prostate cancer cells. More interestingly, Gan et al. demonstrated that TRIM58 is inactivated by promoter methylation in clear cell renal cell carcinoma, and TRIM58 DNA demethylation mediated by CRISPR-dCas9-TET1 can inhibit cancer cell proliferation and migration by reactivating TRIM58 expression.

Secondly, nucleases represented by CRISPR-Cas13 can also be used to cleave or edit cellular RNAs. Since the abnormal expression and activity of the transcriptome are more closely related to diseases, this will be a new direction. Che et al. designed the CRISPR-Cas13a to target the enhancer RNA-SMAD7, and knockdown of SMAD7e inhibits bladder cancer development both *in vitro* and *in vivo*. Zhang et al. also used CRISPR-Cas13 to inhibit the expression of another lncRNA GACAT3, and they found that knockdown of GACAT3 inhibited cell proliferation and motility, and induced apoptosis by increasing p21, Bax, and E-Cadherin expression in bladder cancer. Zhuang et al. used CRISPR-Cas13d, another enzyme similar to CRISPR-Cas13a, to cleave lncRNA MIR497HG and this induces bladder cancer progression through promoting the crosstalk between Hippo/Yap and TGF-β/Smad signaling. Li et al. constructed CRISPR-CasRx to cleave lncRNA LINC00341 which inhibits tumor cell growth both *in vitro* and *in vivo*. More interestingly, Cao et al. designed a dm^6^ACRISPR demethylation system, dCas13b-ALKBH5, to accurately and specifically demethylate 3′UTR of PLOD2 mRNA, which has a high level of m^6^A methylation in renal cell carcinoma. Their results suggested that PLOD2 mRNA demethylated by dCas13b-ALKBH5 might provide a new light on the treatment for renal cell carcinoma.

Thirdly, some intelligent CRISPR systems that regulated by internal and external signals have been designed to treat diseases. Wu et al. presented a blue light-inducible CRISPR-Cas9 system for inhibiting progression of melanoma cells. This light-inducible system may provide a novel strategy for skin cancer treatment. Huang et al. also constructed a light-inducible split-dCas9 system for inhibiting the progression of bladder cancer cells by activating p53 and E-cadherin expression. Zhuang et al. inserted the hTERT aptazyme into 3′UTR of the Cas13d system, and used the reprogrammed system to sense hTERT signals. This may provide a highly effective approach for cancer gene therapy. Yang et al. used CRISPReader technology to construct an endogenous molecular system for sensing the oncogenic transcription factor Ets-1. This system can specifically kill multiple types of cancer cells based on the recognition of Ets-1, and has a wide range of potential applications.

In addition to the works mentioned above, Yao et al. also proposed a special study. They found that synthetic artificial lncRNA shows a higher efficiency in malignant phenotype inhibition compared to the CRISPR/Cas system. This is indeed very interesting, because the guide RNA of the CRISPR-Cas system is very similar to lncRNA in structure and function. Maybe we can build a better gene editing and regulation system based on the artificial lncRNA.

Overall, this Research Topic fully demonstrates the feasibility of the CRISPR-Cas system to treat diseases by sensing or targeting DNA, RNA and protein targets. In the future, the clinical application progress of the CRISPR system will be more worthy of attention.

